# Mapping Failure Mode and Effects Analysis and Other Prospective Risk Analysis Approaches in Intrahospital Transport Involving Critically Ill Patients: A Scoping Review

**DOI:** 10.7759/cureus.119291

**Published:** 2026-11-07

**Authors:** Mario Francesco Galasso, Federico Ruta, Vincenzo Abbasciano, Nunzia Cappucci, Gennaro Laus, Giuseppe Gisario, Valeria Liso, Gianluca Laricchia, Anna Maria Carrozzo, Anna Dora Penta, Alessandro Totaro, Giuseppe Fumai

**Affiliations:** 1 Department of Anesthesia, Policlinic University Hospital, Bari, ITA; 2 Department of Nurse Quality Improvement, National Association of Nurses for Quality and Clinical Risk, Bari, ITA; 3 Department of Human Resources, Local Health Authority Barletta-Trani, Barletta, ITA; 4 Department of Internal Medicine, Lorenzo Bonomo Hospital, Local Health Authority Barletta-Trani, Andria, ITA; 5 Department of Complex Administrative-Legal Framework, Local Health Authority, Foggia, ITA; 6 Department of Services, Scientific Institute for Research, Hospitalization and Healthcare - Referral Cancer Center of Basilicata, Rionero in Vulture, ITA; 7 Department of Emergency, Local Health Authority, Foggia, ITA; 8 Department of Orthopedics and Traumatology, Lorenzo Bonomo Hospital, Local Health Authority Barletta-Trani, Andria, ITA; 9 Department of Gynecological Surgery, Riuniti Hospital, Foggia, ITA; 10 Department of Pediatrics, Local Health Authority, Foggia, ITA; 11 Department of Emergency, Policlinic University Hospital, Bari, ITA; 12 Department of Biomedical Sciences, University of Foggia, Foggia, ITA; 13 Department of Orthopedic and Neurological Rehabilitation, ReHcura Rehabilitation Facility, Adelfia, ITA

**Keywords:** critical care nursing, critical illness, failure mode and effects analysis, healthcare failure mode and effect analysis, intrahospital transport, nursing leadership and management, nursing quality improvement, patient safety, risk management, scoping review

## Abstract

Intrahospital transport exposes critically ill patients and their care teams to clinical and organizational vulnerabilities, and proactive risk analysis can inform preventive interventions. This scoping review mapped the use of failure mode and effects analysis (FMEA), its variants, and complementary prospective risk analysis approaches in intrahospital transport pathways involving critically ill patients or critical care transitions. Studies of multi-area transfers, imaging pathways, and mixed adult and pediatric populations were included for their organizational relevance, not because all participants were critically ill. The review followed the Joanna Briggs Institute (JBI) methodology and was reported in accordance with the Preferred Reporting Items for Systematic Reviews and Meta-Analyses extension for Scoping Reviews (PRISMA-ScR). PubMed, the Cumulative Index to Nursing and Allied Health Literature (CINAHL), Scopus, Web of Science, and the Cochrane Library were searched on October 3, 2026, for studies published from 2002 to 2026. Three reviewers independently screened all records and full-text reports. All included studies were appraised with the Mixed Methods Appraisal Tool (MMAT) 2018, and the six studies with quantitative comparisons were also appraised with the JBI checklist for quasi-experimental studies. Of the 789 records identified, 689 were screened after the removal of 100 duplicates. Fourteen studies, reported in 15 publications, were included. Seven studies used FMEA, five used healthcare failure mode and effects analysis (HFMEA), and one used failure modes, effects and criticality analysis (FMECA); the remaining study used the bow-tie model. Recurring vulnerabilities involved communication and handoff, patient identification, medication management, equipment readiness, device security, and monitoring. Four studies reported fewer adverse events after implementation, one reported improved perceived preparedness, and one reported a lower risk priority number (RPN). Designs and outcomes were heterogeneous. The mapping can inform local risk analysis and the design of interventions, but the reported associations do not establish causal effectiveness. Controlled multicenter evaluations with measures of fidelity, sustainability, and cost are needed. The vulnerabilities and preventive strategies identified are particularly relevant to nursing practice, given the central role of nurses in patient preparation, monitoring, device protection, medication continuity, and interprofessional handoff. No dedicated studies have addressed transport from the intensive care unit or emergency department to the operating room or between intensive care units, and research is needed in underrepresented regions, such as the Middle East, Southeast Asia, Latin America, and most of Europe.

## Introduction and background

Intrahospital transport is the movement of a patient from one area to another within the same hospital for diagnostic or therapeutic purposes. It is a routine and frequent activity; for example, when a patient in the intensive care unit (ICU) is transported for computed tomography, to the operating room or to another unit, and this routine nature tends to mask its risk profile. The risk is high for patients cared for in intensive care or emergency settings, because three unfavorable conditions coexist: clinical instability, dependence on devices that replace a vital function (mechanical ventilation, continuous infusion of vasoactive drugs and invasive pressure monitoring), and a reduced physiological reserve, which makes even a brief disturbance poorly tolerated [1-3].

Transport places the patient in a mobile and changing environment in which each of these conditions can be challenged at the same time. Hemodynamic and respiratory parameters may deteriorate; catheters, tubes and drains may be dislodged; equipment may malfunction or run out of battery power; oxygen or drug delivery may be interrupted; and clinical information may be lost during the handoff between teams [4]. An often overlooked organizational discontinuity adds to these risks: during transport, the patient temporarily leaves the original care setting, with its equipment, protocols, and staff, without yet fully entering the receiving one. The documented consequences range from clinical deterioration [1,2] to complications and a longer stay in the ICU [5], which increases the costs of care, and failures in communication and handoff are a frequent contributing cause of malpractice claims [6].

The literature addresses both the risks of intrahospital transport and the application of failure mode and effects analysis (FMEA) to care processes, and published applications in transport show marked heterogeneity in pathways, methods, and outcomes [1,2,7]. FMEA is a systematic and prospective method [8,9] that was originally developed in industry and later adapted to healthcare. Instead of reconstructing an event that has already occurred, as traditional incident analysis does, it breaks a process down into its elementary steps and asks, for each step, how it could fail before harm occurs. For each failure mode identified, the team analyzes causes and effects, assigns a risk priority score, and develops corrective actions. This score, the risk priority number (RPN), is the product of factors rated on ordinal scales, typically from 1 to 10: the severity of the effect on the patient, the probability that the failure occurs and in classic FMEA, the difficulty of detecting it before it causes harm. The product is used to rank failures by priority for intervention, not to measure risk in a statistical sense.

Several variants have since been developed, including healthcare failure mode and effects analysis (HFMEA), developed by the US Department of Veterans Affairs National Center for Patient Safety [9], and failure modes, effects and criticality analysis (FMECA). The variants differ in the number of steps, in the scoring scales, particularly in whether detectability is included in the calculation, and in the use of support tools such as the decision tree.

Proactive risk analysis is also relevant to patient safety and accreditation frameworks. In Italy, the State-Regions Agreement of December 20, 2012, includes risk management among the accreditation requirements [10], and Article 1 of Law No. 24 of March 8, 2017, recognizes the safety of care as an integral part of the right to health [11]. The Joint Commission International standards include a chapter on quality and patient safety, and the International Organization for Standardization (ISO) 7101:2023 standard sets requirements for quality management systems in healthcare organizations [12-14]. These frameworks support systematic risk management without making FMEA the only applicable technique, and mapping its use in transport links published applications with the needs of quality management systems.

The gap addressed by this review is descriptive and operational. The review determines how many studies were identified, in which settings, which transport phases they analyzed and with which methods, and it examines the recurring vulnerabilities and the derived interventions, distinguishing potentially transferable elements from components that depend on local organization. The heterogeneity of designs, settings and techniques makes a scoping review, complemented by the critical appraisal of all included studies, more appropriate than a synthesis aimed at a pooled estimate of effectiveness.

The objective of this review was to map the evidence on FMEA and its variants, namely, FMEA (including AMFE, the Spanish-language análisis modal de fallos y efectos), FMECA and HFMEA, together with the complementary prospective approaches identified by the search, in intrahospital transport pathways related to critical care, describing settings, populations, methods, risks, interventions and outcomes.

## Review

Methods

Study Design

This scoping review was conducted in accordance with the Joanna Briggs Institute (JBI) methodology for scoping reviews [15] and was reported in accordance with the Preferred Reporting Items for Systematic Reviews and Meta-Analyses Extension for Scoping Reviews (PRISMA-ScR) [16]; the completed checklist is provided in Appendices (part F). The review protocol was not registered or published in advance.

Eligibility Criteria

The eligibility criteria were defined with the Population, Concept, Context (PCC) framework and are detailed in Table 1. The population comprised critically ill adult, pediatric, or neonatal patients and the teams involved in transport, and the unit of interest also included the organizational transfer process; multi-area pathways and hospital transport protocols were therefore eligible, with their organizational relevance distinguished from outcomes specific to critically ill patients. Pediatric and neonatal populations were charted as a distinct subgroup but included in the overall synthesis. The concept was the application of FMEA, AMFE, FMECA, or HFMEA to intrahospital transport, or of another structured method of prospective analysis of the same process, applied before harm occurred to identify vulnerabilities and lead to barriers or corrective actions; these methods are referred to as approaches complementary to FMEA, and the bow-tie model was not classified as an FMEA variant. The context comprised hospitals without geographic or level restrictions, with interunit, diagnostic and perioperative pathways relevant to critical care; prehospital, out-of-hospital and interhospital transport was excluded.

**Table 1:** Eligibility criteria within the Population, Concept and Context framework PCC: Population, Concept, Context; FMEA: failure mode and effects analysis; AMFE: análisis modal de fallos y efectos (Spanish term for FMEA); FMECA: failure modes, effects and criticality analysis; HFMEA: healthcare failure mode and effects analysis

PCC element	Inclusion	Exclusion
Population	Critically ill adult, pediatric and neonatal patients and the teams involved in transport; pediatric and neonatal populations were charted as distinct subgroups. Multi-area processes and hospital transport protocols of organizational relevance to critical care were also eligible and were distinguished from exclusively critically ill samples.	Studies without intrahospital transport or unrelated to the pathways or processes under review; handoff between shifts alone, without an intrahospital transition of the patient, was not sufficient.
Concept	FMEA (including AMFE), FMECA, HFMEA and other structured methods of prospective analysis of the same process that identify vulnerabilities and lead to barriers or corrective actions; the bow-tie model was included and distinguished from the FMEA family. Analysis of the transport process as a whole or of a clearly identifiable phase, including patient preparation, transfer, transport, handoff and repositioning.	Risk analysis applied to other processes without a relevant intrahospital transport component; studies in which transport is mentioned only as a marginal activity or is not the object of the analysis; studies of single clinical procedures unrelated to the transport process.
Context	Hospital settings without geographic or level restrictions: transitions to or from intensive or emergency care, including interunit, diagnostic and perioperative pathways, and hospital transport protocols, including multi-area protocols, described separately if not exclusively critical.	Prehospital, out-of-hospital or interhospital transport, including transfers between different healthcare facilities, in any population.
Types of sources	Any original design with a concrete application of the eligible methods. Publications from 2002 to the search date (October 3, 2026). English or Italian, or another language with a full-text version or a complete abstract in English, verified at screening. A full-text report was required for eligibility assessment and appraisal.	Editorials, commentaries, letters to the editor, opinion articles and conference abstracts without sufficient data; articles whose full text could not be retrieved; gray literature, which was not searched systematically.

Original reports describing a concrete application of the eligible methods were included, whereas editorials, commentaries, letters, opinion articles and conference abstracts without sufficient data were excluded. No language filter was applied, because the indexing language may vary across databases: for example, one included study was indexed in MEDLINE as an English-language article [7], whereas another was indexed as a Spanish-language article despite having English metadata in Scopus [17]. The language criterion was therefore verified at screening.

Search Strategy

Five databases were searched: PubMed, Scopus, the Cochrane Library, the Cumulative Index to Nursing and Allied Health Literature (CINAHL, via EBSCOhost), and Web of Science. The year of publication was the only limit applied. The lower limit of 2002 was chosen because one of the first contributions to introduce FMEA as a means of meeting the proactive risk assessment requirement of the Joint Commission on Accreditation of Healthcare Organizations was published in that year [8].

The strings combined transport and transfer terms with FMEA terms, including acronyms, full names, and spelling variants (such as failure mode effects analysis), supplemented by generic terms for proactive or prospective risk analysis, with fields adapted to each interface. The PubMed and CINAHL strings also included a block on critically ill patients and intensive or emergency settings, whereas the Scopus, Web of Science, and Cochrane strings did not, and the PubMed string included the Medical Subject Headings (MeSH) term Risk Assessment, which broadened retrieval beyond records that name FMEA in the title or abstract. No NOT operator was used; prehospital and out-of-hospital records were excluded at screening. Table 2 reproduces the queries exactly as executed.

**Table 2:** Search strings used in each database The queries are reproduced in full as executed; differences between databases are described in the Methods. No language filter was applied. The 2002-2026 publication year limit is part of the syntax for PubMed, Scopus, and Web of Science and was set through the interface for CINAHL and the Cochrane Library. Database yields are reported in Appendices (part A) and deduplication in Appendices (part E). MeSH: medical subject headings; tiab: title/abstract; TX: EBSCOhost all-text field; TITLE-ABS-KEY: Scopus title, abstract and keyword field; TS: Web of Science topic field; PY: publication year; CINAHL: Cumulative Index to Nursing and Allied Health Literature

Database	Search string
PubMed	( "Transportation of Patients"[Mesh] OR "Patient Transfer"[Mesh] OR "Patient Handoff"[Mesh] OR "intra-hospital transport*"[tiab] OR intrahospital[tiab] OR "in-hospital transport*"[tiab] OR "within-hospital transport*"[tiab] OR "internal transport*"[tiab] OR "patient transfer*"[tiab] OR "interunit transfer*"[tiab] OR "inter-unit transfer*"[tiab] OR handoff[tiab] OR handoffs[tiab] OR "hand-off"[tiab] OR "hand-offs"[tiab] OR handover[tiab] OR handovers[tiab] OR "hand-over"[tiab] OR "hand-overs"[tiab] OR "transfer of care"[tiab] ) AND ( "Critical Care"[Mesh] OR "Intensive Care Units"[Mesh] OR "Emergency Service, Hospital"[Mesh] OR "Critical Illness"[Mesh] OR "critically ill"[tiab] OR "critical patient*"[tiab] OR "critical care"[tiab] OR "intensive care"[tiab] OR ICU[tiab] OR "intensive care unit*"[tiab] OR "emergency department*"[tiab] OR "emergency room*"[tiab] OR "emergency service*"[tiab] OR "emergency-urgency"[tiab] ) AND ( FMEA[tiab] OR FMECA[tiab] OR HFMEA[tiab] OR "Healthcare Failure Mode and Effect Analysis"[Mesh] OR "failure mode and effects analysis"[tiab] OR "failure mode and effect analysis"[tiab] OR "failure modes and effects analysis"[tiab] OR "failure mode effects analysis"[tiab] OR "failure mode effect analysis"[tiab] OR "failure modes effects analysis"[tiab] OR "failure modes effect analysis"[tiab] OR "healthcare failure mode and effect analysis"[tiab] OR "proactive risk analys*"[tiab] OR "prospective risk analys*"[tiab] OR "prospective hazard analys*"[tiab] OR "risk assessment"[Mesh] OR ( "failure mode*"[tiab] AND effect*[tiab] AND analys*[tiab] ) ) AND ( "2002"[Date - Publication] : "2026"[Date - Publication] )
CINAHL (EBSCOhost)	TX ( "intra-hospital transport*" OR "intrahospital transport*" OR "in-hospital transport*" OR "within-hospital transport*" OR "internal transport*" OR "patient transfer*" OR "intra-hospital transfer*" OR "intrahospital transfer*" OR "interunit transfer*" OR "inter-unit transfer*" OR handoff* OR handover* OR "hand-off*" OR "hand-over*" OR "transfer of care" ) AND TX ( "critically ill" OR "critical patient*" OR "critical care" OR "intensive care" OR ICU OR "intensive care unit*" OR "emergency department*" OR "emergency room*" OR "emergency service*" ) AND TX ( FMEA OR FMECA OR HFMEA OR "failure mode and effects analysis" OR "failure mode and effect analysis" OR "failure modes and effects analysis" OR "failure mode effects analysis" OR "failure mode effect analysis" OR "failure modes effects analysis" OR "failure modes effect analysis" OR "healthcare failure mode and effect analysis" OR "proactive risk analys*" OR "prospective risk analys*" OR "prospective hazard analys*" OR ( "failure mode*" AND effect* AND analys* ) )
Scopus	TITLE-ABS-KEY( "intra-hospital transport*" OR "intrahospital transport*" OR "in-hospital transport*" OR "within-hospital transport*" OR "internal transport*" OR "hospital transport*" OR "patient transport*" OR "patient transfer*" OR "interunit transfer*" OR "inter-unit transfer*" OR "intra-hospital transfer*" OR "intrahospital transfer*" OR "transport of critically ill*" OR "transport critically ill*" OR handoff* OR handover* OR "hand-off*" OR "hand-over*" OR "transfer of care" ) AND TITLE-ABS-KEY( FMEA OR FMECA OR HFMEA OR "failure mode and effects analysis" OR "failure mode and effect analysis" OR "failure modes and effects analysis" OR "failure mode effects analysis" OR "failure mode effect analysis" OR "failure modes effects analysis" OR "failure modes effect analysis" OR "healthcare failure mode and effect analysis" OR "proactive risk analys*" OR "prospective risk analys*" OR "prospective hazard analys*" OR ( "failure mode*" AND effect* AND analys* ) ) AND PUBYEAR > 2001 AND PUBYEAR < 2027
Web of Science	TS=( "intra-hospital transport" OR "intrahospital transport" OR "in-hospital transport" OR "within-hospital transport" OR "internal transport" OR "hospital transport" OR "patient transport" OR "patient transfer" OR "patient transfers" OR "intra-hospital transfer" OR "intrahospital transfer" OR "interunit transfer" OR "inter-unit transfer" OR handoff OR handoffs OR handover OR handovers OR "hand-off" OR "hand-over" OR "transfer of care" ) AND TS=( FMEA OR FMECA OR HFMEA OR "failure mode and effects analysis" OR "failure mode and effect analysis" OR "failure modes and effects analysis" OR "failure mode effects analysis" OR "failure mode effect analysis" OR "failure modes effects analysis" OR "failure modes effect analysis" OR "healthcare failure mode and effect analysis" OR "health care failure mode and effect analysis" OR "proactive risk analys*" OR "prospective risk analys*" OR "prospective hazard analys*" ) AND PY=(2002-2026)
Cochrane Library	(transport OR transfer OR "patient transport" OR "patient transfer" OR "hospital transport" OR intrahospital OR "intra-hospital" OR handoff OR handover OR "hand-off" OR "hand-over" OR "transfer of care") AND (FMEA OR FMECA OR HFMEA OR "failure mode and effects analysis" OR "failure mode and effect analysis" OR "failure mode effects analysis" OR "failure mode effect analysis" OR "proactive risk analysis" OR "prospective risk analysis" OR "prospective hazard analysis" OR ( (failure NEXT mode*) AND effect* AND analys* ))

The search was centered on the FMEA family, but its generic terms for prospective risk analysis also retrieved one bow-tie study [18]. The criterion for complementary prospective approaches was specified during screening; after this retrieval, and the study was included because it was consistent with the objective and relevant in terms of population and context; it is described separately from the FMEA studies.

Source Selection

All records were exported as comma-separated values files and imported into Rayyan (Rayyan Systems Inc., Cambridge, MA, USA) [19], where exact duplicates were removed automatically, and potential duplicates flagged by the software were checked manually by title, authors, and year of publication. The unique records were screened by title and abstract against the criteria in Table 1, and potentially eligible reports were retrieved and assessed in full text. A final data verification identified one further duplicate after screening, as described in the Results.

After a calibration exercise, three reviewers (Rev1, Rev2, and Rev3) independently assessed all records and all full-text reports. Disagreements were to be resolved by discussion and, if they persisted, by the arbitration of a fourth reviewer (Rev4). Agreement was described with raw agreement and, where defined, Fleiss' kappa [20], interpreted according to Landis and Koch [21] (Appendices (part D)). The selection process is reported in the PRISMA 2020 flow diagram [22], with the counts for each stage in Appendices (part A) and the full-text eligibility assessment, including the borderline records excluded at screening, in Appendices (part B).

Data Extraction and Charting

The charted data comprised author and year, country, setting, population, design, method (FMEA, AMFE, FMECA, HFMEA or bow-tie), pathway and phases, working group, vulnerabilities and barriers, prioritization, interventions and outcomes. Comparative clinical outcomes, perceived preparedness, RPN values and noncomparative surveillance data were charted as distinct types of outcome. Funding disclosures and potential conflicts of interest of the primary sources were qualitatively reviewed during full-text extraction to monitor for commercial or industrial bias. The screening dataset and the complete charting of each study are provided in Appendices (part E).

Critical Appraisal of Individual Sources of Evidence

Critical appraisal is optional in PRISMA-ScR (item 12, "if done") and in the JBI methodology for scoping reviews [15], but it was performed for all included studies to give readers an explicit picture of the reliability of the reported evidence. The Mixed Methods Appraisal Tool (MMAT), version 2018 [23], was the primary tool because the included designs were heterogeneous. The MMAT comprises two screening questions common to all designs and five criteria specific to each study category, each rated "Yes," "No" or "Can't tell." Studies were assigned to MMAT categories on the basis of their design and primary data rather than the name of the technique alone. Consistent with the MMAT user guide, no overall score was calculated, and the results are presented as criterion-by-criterion judgments with their rationale. The MMAT appraisal was performed independently by Rev1, Rev2 and Rev3, who had also conducted screening and data extraction.

The JBI critical appraisal checklist for quasi-experimental studies [24], which comprises nine items, was used to examine in greater depth the six studies with quantitative comparisons; for the item on the control group, historical controls, parallel groups and single-group designs were distinguished. Rev1, Rev2 and Rev3 appraised the studies independently, and persistent disagreements were resolved by discussion and arbitration by Rev4. A final consistency check, conducted by consensus, applied the same decision rules to equivalent situations across studies; it did not change the number of criteria met by any study (Appendices (parts C1 and C2). No study was excluded on the basis of the appraisal, and the appraisal results were used only to qualify the reliability of the evidence in the synthesis and discussion.

Synthesis and Presentation of Results

No quantitative synthesis (meta-analysis) was performed. The results were synthesized narratively and tabulated by recurring themes: the methodological variants used, the risk areas identified, the interventions derived from the analyses, and the outcomes reported.

Results

Selection of Sources of Evidence

The database searches yielded 789 records: 345 from PubMed, 261 from CINAHL, 77 from Scopus, 75 from Web of Science, 16 from the Cochrane Database of Systematic Reviews (CDSR), and 15 from the Cochrane Central Register of Controlled Trials (CENTRAL). The EBSCOhost search also returned 444 records from the eBook Academic Collection, which was outside the planned sources; because all of them were unique to that collection, they were removed without the loss of any CINAHL record and are not included in the 789 records.

After the removal of 100 duplicates, 689 unique records underwent title and abstract screening. The duplicates include one CINAHL record indexed only under its Spanish title, which had initially been screened and excluded; during data verification, it was identified as a duplicate of an included report [7] and was reclassified accordingly (Appendices (part E)). Screening excluded 674 records: 659 because the concept was not relevant, that is, no proactive or prospective risk analysis method was applied to the intrahospital transport process; 14 because the context or document type was not relevant; and one because the population was not relevant. The remaining 15 reports were all retrieved and assessed in full text, and all were eligible. They correspond to 14 studies, because the study by McElroy et al. was published in two separate reports [2,25]. The selection process is shown in Figure 1.

**Figure 1:**
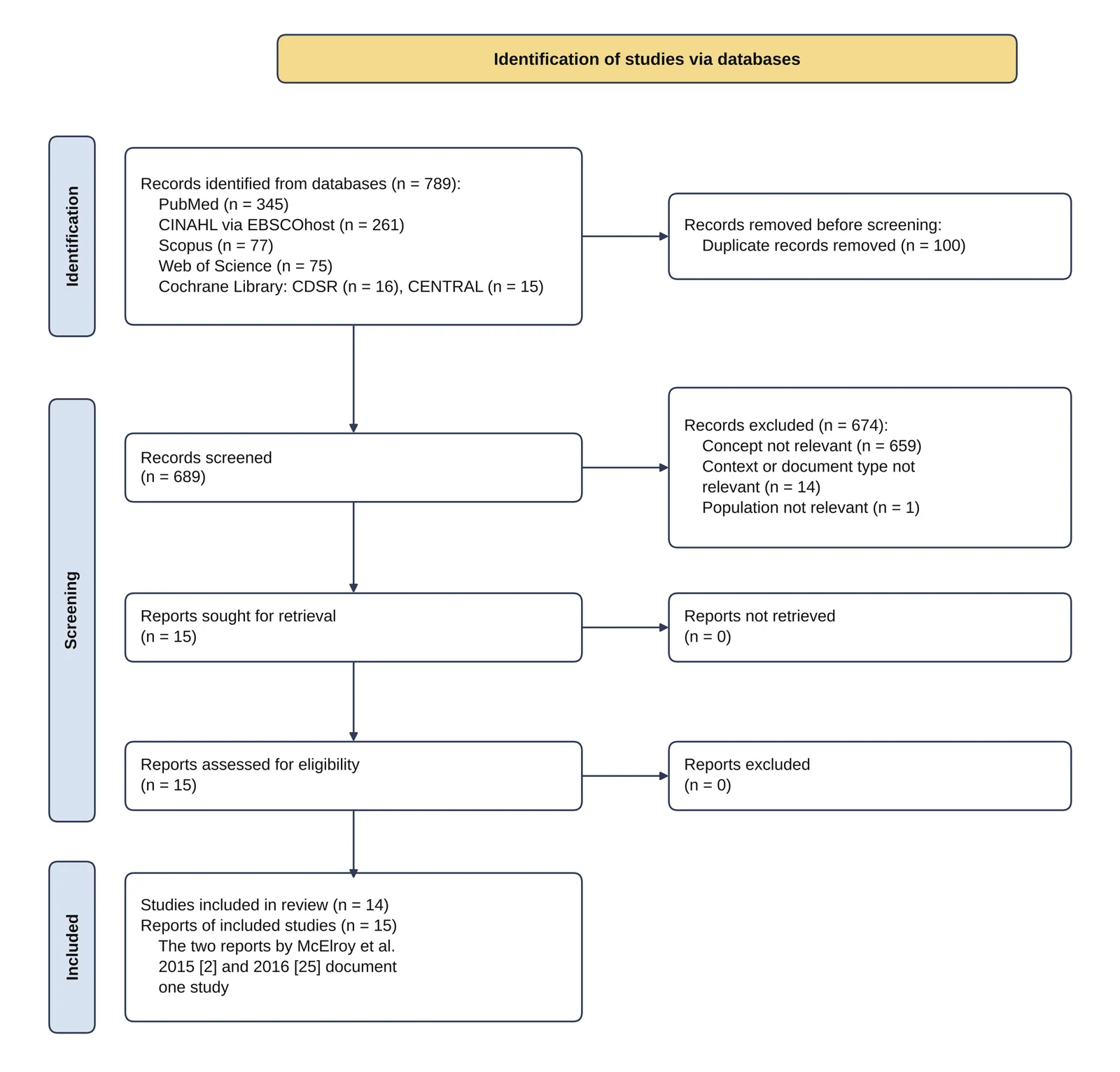
PRISMA 2020 flow diagram of the selection of sources of evidence The PRISMA 2020 flow diagram was adapted to identification through databases only; all searches were run on October 3, 2026. Records, reports and studies are distinct units; the two reports by McElroy et al. (2015 and 2016) document a single study. PRISMA: Preferred Reporting Items for Systematic Reviews and Meta-Analyses; CINAHL: Cumulative Index to Nursing and Allied Health Literature; CDSR: Cochrane Database of Systematic Reviews; CENTRAL: Cochrane Central Register of Controlled Trials

The three reviewers made the same decision on all 689 records at screening (raw agreement, 100%; Fleiss' kappa, 1.000, with a degenerate 95% confidence interval of 1.000-1.000), which does not imply that agreement would be perfect in other samples. At full-text assessment, all reviewers judged all 15 reports eligible (raw agreement, 100%); kappa is undefined when only one category is observed, so only raw agreement is reported. Neither discussion nor arbitration was therefore needed during selection.

Characteristics of Sources of Evidence

The 14 studies, reported in 15 publications from 2013 to 2026, were conducted in the United States (n = 5), China (n = 4), Spain (n = 2), Taiwan (n = 1), Brazil (n = 1) and the Netherlands (n = 1); none was conducted in Italy. Two studies involved pediatric populations [26,27], and Martins et al. [28] included both adults and children; no study focused specifically on neonates. The corpus also includes multi-area processes, coronavirus disease 2019 (COVID-19) protocols and transport for imaging not limited to critically ill patients. The pathways included emergency department (ED) to ward or ICU, operating room to ICU, pediatric cardiac ICU to ward, ICU to diagnostic services and transport for imaging from the examination request to the patient's return.

Classic FMEA or AMFE was used in seven of the 14 studies [7,17,26,28-31], HFMEA in five [1,27,32-34] and FMECA in one [2]. The fourteenth study [18] used the bow-tie model, which does not belong to the FMEA family but falls within the prospective risk analysis methods admitted by the inclusion criteria; it is the only study that organizes the analysis around safety barriers, whether existing or missing, rather than around failure modes. Martins et al. [28] were the only authors to place FMEA within a risk management system structured according to the ISO 31000:2018 standard.

Table 3 summarizes the characteristics of each study, and Table 4 summarizes the main risk areas or failure modes, the interventions derived from the analysis and the outcomes reported. An extended version of Table 4, with outcome values, denominators and methodological notes, is provided in Appendices (part E).

**Table 3:** Characteristics of the 14 included studies (15 reports): settings, populations, pathways and methods FMEA: failure mode and effects analysis; AMFE: análisis modal de fallos y efectos (Spanish term for FMEA); FMECA: failure modes, effects and criticality analysis; HFMEA: healthcare failure mode and effects analysis; ED: emergency department; ICU: intensive care unit; CICU: cardiac intensive care unit; PICU: pediatric intensive care unit; CT: computed tomography; MRI: magnetic resonance imaging; COVID-19: coronavirus disease 2019; ISO: International Organization for Standardization; SQUIRE: Standards for Quality Improvement Reporting Excellence

Author, year	Country	Setting and population	Transport process mapped	Method	Study design
Kerckhoffs et al., 2013 [18]	The Netherlands	Critically ill adults; 28-bed ICU (academic center). No patient sample: multidisciplinary panel of 10 healthcare professionals (4 physicians, 5 nurses and 1 quality department representative)	Intrahospital transport, one of three processes analyzed together with unplanned extubation and communication	Bow-tie (prospective risk analysis)	Prospective descriptive study (multidisciplinary panel)
McElroy et al., 2015 [2]	USA	Adult deceased donor liver transplant recipients; operating room to ICU (tertiary academic hospital). No patient sample: 5 observed OR-to-ICU handoffs and 16 individual and small-group FMECA sessions with surgeons, anesthesiologists, OR and ICU nurses and trainees (number of clinicians not reported)	Operating room-to-ICU handoff	FMECA	Prospective descriptive study (ethnographic observation and interviews), documented in two reports [2,25]
Tan et al., 2015 [29]	China	Acutely critically ill adults; 120 patients transported between January 2011 and December 2012, divided into two groups	Intrahospital transport of acutely critically ill patients	FMEA	Parallel groups (routine care vs. FMEA); allocation reported as random, but procedure not described; appraised as quantitative nonrandomized
Sorrentino, 2016 [30]	USA	Adults; ED to inpatient units. No patient sample: retrospective review of 31 deidentified safety event reports on handoff failures and an FMEA team of 11 multidisciplinary members	ED-to-inpatient unit handoff	FMEA	Quality improvement project led by a clinical nurse specialist (retrospective review of safety event reports and functional block diagram)
Sun et al., 2018 [33]	China	Critically ill patients transferred from the ED or ICU; tertiary hospital (Jiangsu); n = 3,120 (1,488 controls, January-April 2017; 1,632 in the observation group, May-August 2017)	Intrahospital transfer of critically ill patients	HFMEA	Before-after study with a historical control group (January-April 2017 vs. May-August 2017)
Moya Suárez et al., 2019 [17]	Spain	Internal transfers across all clinical areas, including ICU-ED transfers (370-bed hospital). No patient sample: 117 healthcare professionals and 14 nurse managers took part in the analysis	20 types of intrahospital transfer	AMFE (FMEA)	Prospective descriptive study (multidisciplinary working group)
Kulesa et al., 2020 [26]	USA	Pediatric cardiology patients; CICU to cardiology ward (323-bed pediatric hospital). No patient sample: pediatric residents surveyed before (n = 74) and after (n = 23) the intervention	CICU-to-cardiology ward transfer	FMEA, fishbone diagram and key driver diagram	Quality improvement study, pre-post (survey)
Lin et al., 2020 [1]	Taiwan	Mechanically ventilated adults; ICU to CT or MRI (2,300 beds; 161 ICU beds); 3,459 transport sessions (1,019 in 2015, 1,281 in 2016 and 1,159 in 2017), of which 119 were audited	ICU transport for diagnostic examinations	HFMEA	Quality improvement study, pre-post (three phases)
Kwock et al., 2021 [34]	USA	Non-intubated patients with confirmed or suspected COVID-19 transported to and from the OR (academic medical center, Maine). No patient sample: simulated patient; stakeholders from 10 disciplines or services; one tabletop simulation, three path-of-travel runs and two in situ transport scenarios (number of participants not reported)	Intrahospital transport along the pathway defined by the COVID-19 protocol	HFMEA with in situ simulation	Prospective descriptive study: process mapping, tabletop simulation and in situ simulation by an interdisciplinary group
Zhang et al., 2022 [32]	China	Critically ill adults; ED to ICU (teaching hospital; 1,800 beds; 135 ICU beds); n = 112 patients (56 before and 56 after HFMEA implementation)	ED-to-ICU transfer	HFMEA	Action research with a single-group quantitative before-after comparison; reported according to SQUIRE 2.0
Prieto-Molina et al., 2023 [7]	Spain	Adults; ED to inpatient ward (370-bed hospital). No patient sample: working group of 21 professionals (2 physicians, 11 nurses, 4 nursing care assistants, 2 nurse managers, 2 orderlies)	ED-to-general ward transfer	AMFE (FMEA)	Prospective descriptive study (multidisciplinary working group)
Martins et al., 2025 [28]	Brazil	Adults and children; operating room to ICU (tertiary academic center, São Paulo). No patient sample: FMEA team of 13 healthcare professionals; two focus groups of 2-6 participants each	Operating room-to-ICU handoff	FMEA (within ISO 31000:2018)	Prospective qualitative action research with ethnographic observation and pre-post focus groups
Majumdar et al., 2026 [31]	USA	Hospitalized patients on oxygen supplementation undergoing imaging examinations (academic medical center, Newark). No patient sample: multidisciplinary FMEA team analyzing a 28-step transport process	Transport from the imaging request to the patient's return to the referring unit	FMEA	Multidisciplinary process improvement project with monitoring of a safety indicator
Zhang et al., 2026 [27]	China	Critically ill children; pediatric ED to PICU; n = 279 children (160 in the 4 months before and 119 in the 4 months after HFMEA implementation)	Integrated pediatric ED-to-PICU transport	HFMEA	Retrospective before-after study with a historical control group

**Table 4:** Main risk areas, derived interventions and reported outcomes in the 14 included studies Outcomes are reported as stated by the authors of each study; they are not comparable across studies, because designs, units of analysis and denominators differ, and they do not indicate causal effectiveness. Numerical values and denominators are reported in Appendices (part E). ICU: intensive care unit; ED: emergency department; RPN: risk priority number; FMEA: failure mode and effects analysis; VITAL: vital signs, infusions, tubes, alarms and leave; STOP: secretions, tubes, oxygen and power (respiratory therapists) or speak-out, tubes, others and position (radiology technologists); I-PASS: illness severity, patient summary, action list, situation awareness and contingency planning, synthesis by receiver; ISBAR: identify, situation, background, assessment, recommendation; SBAR: situation, background, assessment, recommendation

Author, year	Main risk areas or failure modes	Interventions derived from the analysis	Main outcomes reported
Kerckhoffs et al., 2013 [18]	Critical events in transport; missing but implementable safety barriers	Pre-transport checklist; countersignature after patient handoff (communication process)	No clinical outcome measured; list of barriers and recommendations
McElroy et al., 2015 [2]	Operating room-to-ICU handoff: missed preliminary call, absent team members, equipment malfunction	Automated notifications; communication lead; standard pre-handoff elements	No clinical outcome measured; RPN used to set priorities
Tan et al., 2015 [29]	Failure modes in the transport of acutely critically ill patients and their root causes	Solutions derived from the cause analysis	Lower RPN in the FMEA group; no clinical outcome measured
Sorrentino, 2016 [30]	Mainly communication failures in the ED-to-ward handoff	Standardized handoff; process redesign	No clinical outcome measured
Sun et al., 2018 [33]	Potential failures in the transfer of critically ill patients	Transfer processes optimized with a decision tree	Fewer adverse events and higher satisfaction (comparison with a historical control)
Moya Suárez et al., 2019 [17]	Presurgical communication, shift change, interunit coordination	35 improvement measures (protocols, forms, briefings)	No clinical outcome measured; risk mapping and priorities
Kulesa et al., 2020 [26]	Clinical knowledge, verbal communication, documentation, education	Checklist; transfer algorithm; huddle (discontinued); education	Higher perceived preparedness (self-reported outcome, small sample)
Lin et al., 2020 [1]	Transport preparation and in-transit monitoring	Briefings with reminders (VITAL and STOP); standardization; equipment checks	Lower total RPN; fewer reported adverse events; greater task completeness
Kwock et al., 2021 [34]	Latent safety threats (equipment, team, infection prevention, coordination)	Protocol modified and tested with in situ simulation	No clinical outcome measured; simulation identified additional threats
Zhang et al., 2022 [32]	Failures across five macro-phases (order, preparation, transport, monitoring, handoff)	Three-tier risk-stratified protocol; information system; team and equipment defined for each tier	Fewer adverse events per transported patient
Prieto-Molina et al., 2023 [7]	Patient and allergy identification; medication administration	Admission checklist; transfer protocol; training	No clinical outcome measured; process outputs (protocol and checklist)
Martins et al., 2025 [28]	Missing or incomplete information between teams; preanesthetic assessment; transfer of responsibility for the patient	23 actions (I-PASS or ISBAR, training, mobile equipment, bed logistics)	No clinical outcome measured; qualitative pre-post assessment of risk awareness
Majumdar et al., 2026 [31]	28 steps of transport for imaging; five prioritized	Action plan for each priority step; updated policies and algorithms	No serious safety events reported (surveillance without a baseline; not an effect estimate)
Zhang et al., 2026 [27]	Equipment readiness, lines and vascular access, communication and handoff, medications, monitoring	Handoff checklist; equipment standardization; SBAR; periodic multidisciplinary reviews	Fewer incidents than in the historical control; higher satisfaction with collaboration

Critical Appraisal Within Sources of Evidence

The MMAT was applied to all 14 studies, and all of them met the two screening questions. Two studies were appraised as qualitative (category 1): McElroy et al., with its additional 2016 report [2,25], and Martins et al. [28]. Six were appraised as quantitative nonrandomized (category 3): Tan et al. [29], Sun et al. [33], Kulesa et al. [26], Lin et al. [1], Zhang et al. [32] and Zhang et al. [27]; Tan et al.' study was classified as nonrandomized because the allocation, although reported as random, was insufficiently described. Six were appraised as quantitative descriptive (category 4): Kerckhoffs et al. [18], Sorrentino [30], Moya Suárez et al. [17], Kwock et al. [34], Prieto-Molina et al. [7] and Majumdar et al. [31].

Among the qualitative studies, McElroy et al. met all criteria, and Martins et al. met four of five, because the risk-scoring phase was not described in sufficient detail. In the six quantitative descriptive studies, the sampling strategy was rated appropriate in five (criterion 4.1) and the statistical analysis in all six (criterion 4.5), whereas the appropriateness of the measurements and the risk of nonresponse bias (criteria 4.3 and 4.4) were rated "Can't tell" in all six. Among the quantitative nonrandomized studies, Lin et al. met four of five criteria, Sun et al. three and Zhang et al. two, whereas "Can't tell" or unmet criteria predominated in Tan et al., Zhang et al. and Kulesa et al. No study met the criterion on confounders (criterion 3.4), which was rated "Can't tell" in four studies and "No" in Kulesa et al. and Zhang et al., whose reports document organizational or structural changes coinciding with the intervention that the analysis did not account for. The MMAT judgments of the three reviewers were unanimous for all criteria, and the complete grids, with their rationale, are provided in Appendices (part C2).

With the JBI checklist, applied to the five before-after comparisons and to the parallel-group study by Tan et al., Kulesa et al. met three of eight applicable items, Lin et al. met eight of nine, Zhang et al. met four of nine (four rated unclear), Zhang et al. met six of nine (one rated unclear), Tan et al. met six of nine (two rated unclear), and Sun et al. met six of nine (two rated unclear). These are descriptive counts of criteria, not validated quality thresholds; the individual judgments and the resolution of disagreements are reported in Appendices (part C1).

Methodological Variants and Prioritization

The FMEA studies shared process reconstruction, failure identification and the definition of actions, but they did not all quantify risk in the same way. Prieto-Molina et al. [7] and Moya Suárez et al. [17] used severity, probability and detectability, with an action threshold of 100, and Prieto-Molina et al. also proposed actions for five failures below the threshold, whereas McElroy et al. [2] used FMECA with a threshold of 300. The HFMEA applications [1,27,32,33] used scores and decision rules reported in varying detail. Kulesa et al. [26] combined FMEA with a fishbone diagram and a key driver diagram, and Kwock et al. [34] combined HFMEA with simulation. The bow-tie analysis by Kerckhoffs et al. [18] identified threats, consequences and barriers without an RPN, whereas Tan et al. [29] used the RPN as an outcome compared between groups. The indices were therefore not compared directly.

The second report of the study by McElroy et al. [25] is the only contribution in the corpus that tested the prioritization method empirically rather than simply applying it. In this 2016 report, 79 failures identified in the 37 steps of the operating room-to-ICU handoff were ranked twice: with the traditional method, based on the RPN and the criticality index, and with a simplified method that classifies each failure as high, medium or low risk. Of the 24 failures with an RPN of 300 or higher, 22 were also classified as high risk by the simplified method, an agreement of 92%; however, agreement was 50% (6 of 12) for the failures in the top 20% of the criticality index, and the authors concluded that the simplified method discriminates less finely between failures than the traditional method. The failure counts of the two reports (81 in 2015 and 79 in 2016) were not summed.

Recurring Risk Areas

Despite the heterogeneity of settings, the priority risk areas converged across the 14 studies. The first was communication and handoff between teams, which was often incomplete, delayed or not standardized [2,17,26-28,30,32]; in the study by Martins et al. [28], missing or incomplete information at team interfaces and shift changes was the failure mode with the highest criticality. The other areas were patient identification and verification of clinical documentation [7,17,33]; medication management, particularly medication reconciliation before transfer [7,17]; the readiness and functioning of transport equipment, such as monitors, ventilators, oxygen sources and batteries [1,2,18,27,32]; the security of devices and lines, such as venous access, endotracheal tubes and drains [7,27]; the composition and preparation of the transport team, including the absence of qualified staff or poorly defined roles [1,17,32,34]; continuous clinical monitoring during transport and the prompt detection of deterioration [2,18,27,32]; and coordination with the receiving unit and with diagnostic services, from the examination request to the patient's return to the unit [30,31,33].

Derived Interventions and Reported Outcomes

Checklists were described by Prieto-Molina et al. [7], Kulesa et al. [26] and Zhang et al. [27] and were recommended by Kerckhoffs et al. [18], and Moya Suárez et al. [17] proposed standardized documentation tools. Other interventions included structured handoff tools, namely, the situation, background, assessment and recommendation (SBAR) technique [27] and the I-PASS or ISBAR handoff mnemonics [28]; briefings supported by mnemonic reminder cards (VITAL and STOP) [1]; algorithms with defined roles [2,26]; and risk-stratified transport [32]. Kwock et al. [34] tested the protocol in simulation, and education accompanied several projects. Proposed interventions were not considered evidence of implementation or effectiveness.

In the studies that measured clinical outcomes before and after implementation, the authors reported, within their own designs, a statistically significant reduction in the incidence of transport-related adverse events: from 19.64% to 7.14% (Zhang et al. [32], p < 0.05), from 1.08% to 0.23% (Lin et al. [1], p = 0.01), from 35.0% (56 incidents in 160 children) to 6.7% (8 incidents in 119 children) (Zhang et al. [27], p < 0.001) and from 22.58% to 5.94% (Sun et al. [33], p < 0.001). The study by Sun et al. included 1,488 control and 1,632 observation patients, the largest sample in the corpus, and also reported an increase in patient satisfaction from 77.96% to 95.96% and in the satisfaction of receiving departments from 68.95% to 94.98% (both p < 0.001).

These four reductions are not comparable with each other, because they rest on different denominators: transported patients (Zhang et al. [32]), transport sessions (Lin et al. [1]), children transferred from the pediatric ED to the PICU, with incidents summed across four event categories and a historical control (160 vs. 119 children; Zhang et al. [27]) and patients in the two periods (Sun et al. [33]). Because a patient may be transported several times during a hospital stay, the same number of events produces different percentages depending on the unit of analysis. The four values therefore show a concordant direction but cannot be compared in magnitude, and they are descriptive data from individual studies, not a review-level estimate of the effectiveness of FMEA or HFMEA.

Kulesa et al. [26] reported an improvement in residents' perceived preparedness at the time of transfer, from 15% before to 83% overall after the intervention, although with a small postintervention sample (n = 23, with 8-15 responses per item). Tan et al. [29] reported a lower RPN in the experimental group than in the control group but measured no clinical outcome, so the reduction concerns the score assigned to the process, not patient harm. Majumdar et al. [31] reported no serious safety events throughout 2020 and up to July 2021, a surveillance finding without an explicit baseline from the preceding period. The descriptive studies [2,7,17,18,28,30,34] mapped risks and proposed corrective actions without a quantitative assessment of clinical outcomes; Martins et al. [28] also assessed process risk awareness among the professionals involved before and after the intervention, using focus groups and similitude analysis.

Discussion

This review mapped 14 studies, reported in 15 publications, that applied FMEA, its variants or, in one case, the bow-tie model to intrahospital transport and to transitions relevant to critical care. The corpus is small and geographically concentrated, with eight of the 14 studies published from 2020 onward, and the mapping is not a census of all local applications.

The risk areas identified converged across settings. Communication and handoff, patient identification, medication management, equipment readiness and device security emerged repeatedly as critical points, regardless of the setting (ED, operating room or ICU) and the population (adult or pediatric). This suggests that intrahospital transport involving critically ill patients or critical care transitions shares a common core of process vulnerabilities, although two cautions apply. The first is methodological: FMEA is a team-driven technique, and teams composed according to similar criteria in hospitals with similar organizations tend to produce similar maps, so the recurrence may partly reflect the structure of the method rather than only the structure of the risk. The second concerns the independence of the data: two of the 14 studies [7,17] come from the same 370-bed hospital and share part of the research team, so the mapping represents 13 independent organizational settings rather than 14. Nevertheless, the convergence persists among studies that share neither health system, language nor methodological variant, and this is the main argument for a common core of vulnerabilities.

The operating room-to-ICU transition allows a comparison of two independent studies published 10 years apart on different continents: McElroy et al. [2] in the United States and Martins et al. [28] in Brazil. Despite different approaches, both identified missing or incomplete preliminary communication between the two teams as the failure with the highest criticality, and both recommended designating a communication lead and adopting standardized information elements. Martins et al. [28], however, used FMEA as an analysis technique within an ISO 31000:2018 risk management system, with documented phases of communication, context definition, assessment, treatment, and monitoring. As a result, the working group also ranked risks to staff, to the reputation of the organization, and of an economic nature, and it defined the monitoring phase, with responsibilities and reassessment frequencies, from the outset. This provision, however, is not empirical evidence that the interventions are sustained.

These findings fall within a known trajectory. The risk areas identified in the included studies largely coincide with those described in the literature on clinical handoffs, which documents the consequences of inadequate handoffs and reports communication and environmental barriers to the transfer of information [35]; in that literature, standardized handoff programs have been associated with fewer medical errors and preventable adverse events [36]. The contribution of the included studies is therefore not the identification of new risks but their explicit ranking: FMEA assigns an order of priority and allows resources to be directed to the most critical failures rather than distributed uniformly. Similarly, the criticism of the RPN as an ordinal measure treated as if it were an interval measure, raised by McElroy et al. [2] within the included corpus, reflects an established debate in the methodological literature on FMEA [37,38]. The product of three ordinal scores has no metric properties [37], so an RPN of 400 does not describe twice the risk of an RPN of 200, and scores from differently calibrated scales are not comparable across studies. This property, together with the heterogeneity of designs and outcomes, supports the decision not to perform a quantitative synthesis.

Most studies with a quantitative assessment of outcomes used a nonrandomized, single-center before-after design of variable duration. Although such designs are consistent with the quality improvement nature of these projects, they cannot exclude confounding factors such as seasonal changes in case mix, concurrent staffing changes or other safety initiatives launched in the same period, as the authors of several included studies acknowledged. In a letter on the application of HFMEA to pediatric intrahospital transport, excluded at screening because of its document type and cited here only as commentary, Damasco et al. [39] raised the same caution against attributing to the intervention the improvements observed in single-center before-after designs without a concurrent control.

Sustainability was assessed unevenly. Kulesa et al. [26] measured perceived preparedness at 4 and 18 months (88% and 80%, with 8 and 15 respondents, respectively, versus 15% before the intervention); these numbers do not allow a decline in effect to be inferred, but the discontinuation of the huddle and variable adherence were documented. Lin et al. [1] combined annual phases from 2015 to 2017 with a g-chart (a statistical process control chart for rare events) of reported events from 2013 to 2018, which provides longitudinal monitoring despite the limitations of incident reporting and convenience audits. Organizational cost, fidelity and the durability of benefit require dedicated measures.

Implications for Practice and Quality

The mapping can inform the use of checklists, structured communication, equipment checks and risk-stratified protocols, with roles, communication and equipment as a local starting point to be adapted and evaluated. The included studies did not assess cost systematically, and cost should not be assumed to be low for all interventions. In Italy, the findings on medication reconciliation can be read alongside recommendation no. 17 of the Ministry of Health [40]. Proactive risk management is consistent with quality management systems and accreditation, but the choice of technique should be justified in relation to the process and the available resources.

The review offers a starting framework for multiprofessional teams without indicating the general superiority of classic FMEA, HFMEA or barrier-based methods. Scales, thresholds and decision rules should be explicit and complemented by clinical judgment. For nursing, the operational areas are patient preparation, the continuity of medication and monitoring, device protection and handoff; adherence to these measures and the related workload should also be monitored.

Research Gaps

The gaps include the scarcity of multicenter studies and concurrent controls, the inconsistent reporting of scales and thresholds, the absence of studies specific to neonates and the small number of pediatric studies that addressed transport from the pediatric ED to the pediatric ICU [27] and from the cardiac ICU to the ward [26]. Measures of workload, cost and fidelity are also needed. Lin et al. [1] reported shorter time-outs (for ICU nurses, from 41.6 ± 10.9 to 27.1 ± 9.9 seconds), a temporal process measure that does not quantify the overall additional workload, and the authors stated that the costs and the implementation burden of the strategies were not measured.

The pathways covered include operating room to ICU, ED to ward or ICU, pediatric cardiac ICU to ward and ICU to diagnostic services. Because multi-area analyses were included, the absence of individual pathways cannot be inferred from the overall synthesis, but dedicated and comparable descriptions of ICU-to-operating room, ED-to-operating room and inter-ICU transfers, with explicit phases, responsibilities and populations, would be useful.

The corpus covers six geographic settings and includes no Italian study. No studies were identified from the Middle East, Southeast Asia, Central America or the Caribbean; South America was represented by a single study, and Europe by two countries. Within the databases searched, the absence of Italian studies does not allow a gap in implementation to be distinguished from a gap in dissemination: local risk management projects may be documented in gray literature or in the internal reports of hospital and regional quality systems, within the framework of the 2012 State-Regions Agreement [10], rather than in indexed journals. Because gray literature was not searched systematically, this review cannot verify this hypothesis. National clinical risk management networks could establish open-access registries for sharing local proactive analyses, so that valuable patient safety data do not remain isolated within individual healthcare organizations.

Strengths and Limitations

This review has several strengths. All records and full-text reports were assessed independently by three reviewers, with agreement reported and the decisions of each reviewer provided in the Appendices. All 14 studies were appraised with the MMAT, with judgments and rationale reported for each criterion, and the six quantitative comparisons were further examined with the JBI checklist. The complete search strings, the search date and the yield of each database are reported, so the search can be reproduced. Clinical outcomes, perceived preparedness, RPN values and noncomparative surveillance were kept distinct, with their units of analysis and available denominators.

This review also has limitations. First, terminology varies: the queries included acronyms and alternative names of the method, but contributions that use different or translated terminology may have been missed, and controlled vocabularies do not eliminate the dependence on indexing.

Second, five databases were searched, and gray literature was not searched systematically. No language filter was applied, but differences in indexing and in the availability of English versions may still limit completeness. Indexing also affected deduplication: one CINAHL record indexed only under its Spanish title was not recognized as a duplicate until data verification and had been excluded by all three reviewers, which shows that complete agreement between reviewers does not guarantee accuracy.

Third, the architecture of the search differed across databases. The PubMed and CINAHL strings combined three blocks with AND, whereas the Scopus, Web of Science and Cochrane strings combined only transport and method. To ensure maximum clinical sensitivity and avoid the accidental omission of poorly indexed studies, no NOT operators were deliberately used to exclude prehospital transport during the database execution; instead, these records were transparently managed and excluded during the manual screening phase. Furthermore, the search focused on the FMEA family, and the criterion on complementary prospective approaches was specified during screening; the retrieval of a bow-tie study does not demonstrate exhaustive coverage of all prospective techniques, although its separate analysis confirms the thematic consistency of the core findings.

Fourth, the included organizational contexts are not fully independent, and the data were not synthesized quantitatively. Two of the 14 studies originated from the same center with overlapping research teams, and one study is documented by two separate reports. Consistent with scoping review methodology, no meta-analysis was performed, particularly because the extreme heterogeneity of the risk-scoring scales prevents a direct quantitative comparison of priority scores across different hospital settings. Finally, while the specific funding streams of the primary studies were not aggregated into a separate quantitative table, the full-text review confirmed that the corpus consists of independent, investigator-initiated quality improvement projects funded through institutional or hospital resources, with no commercial sponsors reported.

## Conclusions

This scoping review mapped 14 studies, reported in 15 publications, on the use of FMEA, its variants and one complementary prospective approach in intrahospital transport and in transitions relevant to critical care. Recurring vulnerabilities involved communication, patient identification, medication management, equipment, devices and monitoring. Four studies reported fewer adverse events, one better perceived preparedness and one a lower RPN, but the heterogeneity of designs, populations and units of analysis precludes a pooled causal estimate. The mapping can guide local risk analysis, although it does not replace an assessment of the local context or demonstrate the superiority of one technique over another. Controlled multicenter studies with measures of fidelity, sustainability and cost are needed.

## Appendices

Appendices are openly available at: https://doi.org/10.5281/zenodo.23941752

Part A: PRISMA 2020 selection flow and database yields. Part B: Full-text eligibility assessment. Part C: Critical appraisal (C1, JBI checklist for quasi-experimental studies, six studies; C2, MMAT 2018, 14 studies). Part D: Inter-reviewer agreement. Part E: Screening dataset (689 unique records), removed duplicates, included reports, data charting of the 14 included studies, and the manuscript reference list. Part F: Completed PRISMA-ScR checklist.

PRISMA-ScR: Preferred Reporting Items for Systematic Reviews and Meta-Analyses extension for Scoping Reviews; JBI: Joanna Briggs Institute; MMAT: Mixed Methods Appraisal Tool
